# The impact of sample selection strategies on genetic diversity and representativeness in germplasm bank collections

**DOI:** 10.1186/s12870-019-2142-y

**Published:** 2019-11-27

**Authors:** Jorge Franco-Duran, José Crossa, Jiafa Chen, Sarah Jane Hearne

**Affiliations:** 1 Facultad de Agronomía, Universidad de la República and CIMMYT, Ruta 3, Km 363, 60000 Paysandú, Uruguay; 2 CIMMYT, KM 45 Carretera Mexico-Veracruz, El Batan, 56237 Texcoco, Edo. De Mexico Mexico; 3 Present Address: Henan Agricultural University, No. 95 Wenhua Road, Zhengzhou, 450002 Henan China

**Keywords:** Sampling Genebanks accessions, Genetic diversity representativeness, Sample size, Independent and nested samples, SNPs

## Abstract

**Background:**

Germplasm banks maintain collections representing the most comprehensive catalogue of native genetic diversity available for crop improvement. Users of germplasm banks are interested in a fixed number of samples representing as broadly as possible the diversity present in the wider collection. A relevant question is whether it is necessary to develop completely independent germplasm samples or it is possible to select nested sets from a pre-defined core set panel not from the whole collection. We used data from 15,384, maize landraces stored in the CIMMYT germplasm bank to study the impact on 8 diversity criteria and the sample representativeness of: (1) two core selection strategies, a statistical sampling (DM), or a numerical maximization method (CH); (2) selecting samples of varying sizes; and (3) selecting samples of different sizes independently of each other or in a nested manner.

**Results:**

Sample sizes greater than 10% of the whole population size retained more than 75% of the polymorphic markers for all selection strategies and types of sample; lower sample sizes showed more variability (instability) among repetitions; the strongest effect of sample size was observed on the CH-independent combination. Independent and nested samples showed similar performance for all the criteria for the DM method, but there were differences between them for the CH method. The DM method achieved better approximations to the known values in the population than the CH method; 2-d multidimensional scaling plots of the collection and samples highlighted tendency of sample selection towards the extremes of diversity in the CH method, compared with sampling more representative of the overall genotypic distribution of diversity under the DM method.

**Conclusions:**

The use of core subsets of size greater than or equal to 10% of the whole collection satisfied well the requirement of representativeness and diversity. Nested samples showed similar diversity and representativeness characteristics as independent samples offering a cost effective method of sample definition for germplasm banks. For most criteria assessed the DM method achieved better approximations to the known values in the whole population than the CH method, that is, it generated more statistically representative samples from collections.

## Background

Germplasm banks globally maintain national and international collections of the world’s most important food and forage species for the benefit of humanity. Together these collections make up the most comprehensive catalogue of native genetic diversity offering a valuable underexplored resource for crop improvement in the face of challenges of population growth, climate change, changing diets etc. [1]. In spite of the inherent value of these collections, many germplasm bank clients face a daunting task when trying to select appropriate materials for their particular use case. The sheer number of collections and the sparse passport and characterization data often available make selection challenging.

To address some of the challenges of intelligible selection of accessions from germplasm banks, a number of initiatives have employed next generation sequencing and genotyping to more comprehensively characterize some aspects of the diversity of collections. The maize and wheat focused “Seeds of Discovery” initiative (https://seedsofdiscovery.org/) and the rice focused “3000 genomes” project (http://iric.irri.org/resources/3000-genomes-project) are two examples aiming to study the vast diversity stored in maize, wheat and rice germplasm banks. This genomic characterization, either alone or in combination with other data resources, offers a new lens on germplasm bank collections, potentially facilitating more user-relevant germplasm selections to be made. Despite the immense value of this data resource, clients typically cannot evaluate or utilize all materials of interest and some form of sub-setting of either the collection as a whole or components of interest needs to be conducted. The notion of representativeness is important in this context and its quantification under different practical methods is relevant for maintaining the genetic base of samples taken from the overall germplasm bank collection. The representativeness being particularly relevant when selecting materials for evaluation and potential breeding as to avoid bottlenecks that rapidly constrain genetic variability. Genetic markers have over the years been deployed as sources of information which can be used to assess representativeness of germplasm samples in genetic conservation activities such as accession regeneration and collection.

Traditionally genetic resources stored in ex-situ germplasm banks have been sampled with the objective of forming core subsets for conservation purposes and for studying genetic diversity of accessions stored in germplasm banks. Core subsets (or core samples) can be formed on the basis of morphological, phenotypic, or molecular marker data and are assembled to facilitate the study, evaluation, and utilization of genetic resources stored in ex-situ germplasm collections [2–4]. Core subsets typically include 5 to 20% of the total number of accessions [3–8] and thus a core subset is expected to represent a reduction in the genetic and phenotypic diversity available compared with that in the original collection. In genetic resource conservation, the formation of core collections and/or core subsets is of paramount importance to preserve in the core as much as possible of the diversity present in the original collection (representativeness). Commonly a germplasm bank client with interests outside of the conservation arena (e.g., a plant breeder or a molecular geneticist) is not interested in a fixed proportion of a collection, i.e., a classic core subset; rather she/he is more interested in a fixed number of “representative samples” which may possess characteristics of value, for example represent a particular geographic region, adaptation or originate where a particular stress is prevalent. These “breeder subsets”, like core subsets, need to represent as broadly as possible the diversity present in the wider collection.

Given these needs, a sampling strategy should define both a sampling method and an allocation method [9]. A stratified sampling strategy suggests first classifying the accessions into non-overlapping groups (or clusters) and then a method for allocating accessions in the cluster into the sample. In late 90s and early 2000s, intense research was published on sampling and allocation strategies as well as methods for forming core subsets [10–14]. These authors proposed a sequential clustering strategy for forming core samples using discrete and continuous morphological data simultaneously. The main idea was to initially form groups (clusters) using a geometric method such as the Ward method (which minimizes the variance within a group). Then a “mixture of normal distributions” statistical method acts on the previous clusters by changing the shape, direction and volume of the groups, maximizing the likelihood function and determining the probability of each accession belonging to each group. This two-stage classification approach was used by [15] for forming diverse core subsets of several landraces of tropical maize (*Zea mays* L).

In terms of the allocation strategy for sampling the cluster when using a mixture of continuous and discrete (categorical) phenotypic traits, sampling could be constant across clusters, proportional to the number of individuals in the cluster or proportional to the distance between accessions within a cluster, such Gower’s distance, the D allocation method [16]. The D allocation method of sampling can also be applied with molecular markers so that the sample drawn from each group should be proportional to the genetic distance or allele diversity within each cluster [17]. Ergo, a final subset will represent proportionally more individuals from a genetically diverse cluster than it will from a less genetically diverse cluster, regardless of the total number of individuals within each cluster. This strategy ensures good representativeness of the collection and a high allele richness in the core sample, while the application of genetic distance provides a Euclidean representation of genetic distance.

In terms of modern genomic-enabled prediction accuracy on germplasm bank accession, [18] studied the prediction accuracy of core samples obtained from 8416 Mexican wheat landraces and 2403 Iranian wheat landraces stored in germplasm banks. The authors defined 10 and 20% core samples based on two criteria. One criterion was the reliability measures related to the prediction error variance that was taken as the objective function to be minimized by applying a method that used the first 100 principal components of the marker data [19]; they were called, in the study of [18], predictive core samples. The other criterion for selecting the 10 and 20% core samples was based on the D method of [16] using the Modified Rogers’ genetic distance between pairs of accessions. The final analysis of genomic-enabled prediction accuracy in this study indicated that the use of 10% or 20% cores did not adversely impact the prediction accuracy of traits compared with the whole sample, further supporting that the diverse core samples formed maintained sufficient diversity and representativeness of the population under study.

Several other strategies have been studied and proposed for maintaining allele richness in core samples. One effective strategy maximizes the number of alleles at each marker locus; this is the M strategy [5]. Another strategy maximizes the number of alleles in the core samples by sampling accessions from groups in proportion to within cluster genetic diversity. Furthermore, other strategies for forming core samples attempt to maximize the allele diversity in the core samples, whereas other methods maximize the representativeness of the genetic diversity in the core samples [20]. On the other hand, other methods avoid selecting similar accessions at the extreme of the collection thus maximizing the average distance between each accession and the closest other accession in the core [21].

Authors in [22] studied several formulas for calculating the specificity of the different marker alleles with reference to their distribution across accessions; for assessing the accession rarity based on the specificity of its alleles; for calculating divergence as defined by the Kullback-Leibler formula; for estimating the allele richness in the whole collection; and for computing the lost alleles (lost alleles that are not in the core sample), as well as the Shannon diversity index. These formulas as well as the Modified Roger genetic distance (MR) were used with the HCore and other strategies for forming core subsets (REMC, MixRep, MSTRAT, and random sample) [23]. The above mentioned authors [22] applied these formulas and methods to a large wheat collection. For 10% core samples, the Kullback-Leibler criterion was slightly superior (0.442) to the MR genetic distance (0.438) but the MR overcame the divergence for the other methods. For 20% core samples, the Kullback-Leibler criterion was the same as the MR genetic distance (0.434) but superior to the other methods. Useful approaches using the Kullback-Leibler are (i) determining accession rarities based on the average specificity of their alleles, (ii) ranking alleles according to their specificities, and detecting alleles that are common in only some accessions, and (3) ranking the accessions by their rarity and divergence, thus detecting a group of rare and specific accessions that may have certain potential for important phenotypic traits.

Another method for forming diverse core samples of different sizes proposes a pseudo-index for integrating genetic distance and diversity indices [24], this index serves as a means of optimizing more than one genetic measure simultaneously based on weights assigned to standard measures. The mentioned authors [24] proposed the Core Hunter (CH) algorithm that uses an advanced stochastic local search algorithm to maximize the pseudo-index and show results that are slightly better than the performance of the D-method for several diversity indices (see Table 1 of [24]), but only when a single measure is being optimized. Recently an improvement of the initial Core Hunter (Core Hunter 1 and 2), Core Hunter 3 (CH3) from [25] included two methods for summarizing distances, entry-to-nearest-entry and accession-to-nearest entry proposed by [21]. In addition, CH3 incorporated two new, improved methods for summarizing distances to quantify diversity or representativeness of the core collection and is more effective at maximizing the improved diversity metric than Core Hunter 1 and 2.

Given the high dimensionality of the problem of selecting the core subsets and the largest possible number of different core samples, the problem has been approached in multiple ways and today the solutions and proposals can be divided into two main methodological approaches: (1) the statistical approach using the basic concept of “stratified random sampling selection” with the D allocation method, and (2) the numerical-algorithmic approach using the basic concept of “numerical maximization approach” such as that used by Core Hunter (CH3). Both of them focus on the same objective: obtain a sample containing most of the genetic diversity present in the collection, but the former is based on the statistical concept of the representativeness of a random sample (particularly, the representativeness of the core genetic diversity, and of course, their different measures), while the latter is based on the mathematical concept of the selection of a subset maximizing some criteria (one or more of the measures used for describing the genetic diversity).

For both approaches (D-allocation method or Core Hunter), two principal questions arise when selecting samples for germplasm bank managers or germplasm bank clients. The first question is: what is the minimum sample size needed to optimally represent the diversity of either the whole collection or that fraction of the collection of particular value (e.g., a particular race, species, etc.)? This question is a shift from the classic 10–20% of the collection approach for defining germplasm sets. This area of inquiry is of particular relevance as genome re-sequencing costs continue to decrease and germplasm bank clients begin to ask how many and which accessions should be sequenced to capture the most variation. The second question reflects a growing demand from germplasm bank clients to obtain a set number of entries, e.g., 150 accessions that are diverse and representative. In this case, from the perspective of collection managers, it is relevant to ask if it is necessary to develop completely independent germplasm sets or whether it is possible to form nested sets in such a way that sampling for a panel is done not from the whole collection but a large pre-defined panel. In this case, a nested system is simple maintaining sufficient reserves of seed/clones for distribution, with the benefit that clients could potentially crowdsource evaluation data to build a wealth of knowledge around a common set of accessions.

Based on the above consideration, the objective of this paper is to evaluate, using data from over 15,000 maize landraces stored in the CIMMYT maize germplasm bank, the impact on diversity and representativeness of (1) selecting samples of sizes 5, 10, 20, 30, 40 and 50% from the whole collection (the sample size effect), (2) the influence of independent versus nested sampling of a collection (the sample method effect), and (3) the relative merits of employing either a statistical sampling strategy represented by the D-method with MR genetic distance or a numerical maximization method represented by CH3 and MR distance (the strategy method).

## Results

### Definition of sampling in the D-method (DM)

As already described, the DM-method is a 3-stage method: first a classification (clustering) is done, then the proportion of accessions to be selected from each cluster is defined proportionally to the cluster diversity (measured by the group *mrd* average value), and finally, the best (most diverse) sample out of a thousand candidate samples generated by stratified random sampling process is selected (Table 1).

**Table 1:** Assignation of sample size following the DM-method

Cluster	N	*mrdMean*	*p*	*nD*	s50^a^	s40	s30	s20	s10	s05
1	3042	0.1104	0.1710	1316	1328	1053	789	526	263	132
2	4144	0.1100	0.1703	1310	1322	1048	786	524	262	131
3	2956	0.1066	0.1651	1270	1270	1016	762	508	254	127
4	1408	0.1189	0.1842	1417	1407	1134	850	567	283	142
5	2772	0.1094	0.1695	1304	1304	1043	782	522	261	130
6	1062	0.0902	0.1397	1075	1061	860	645	430	215	107
Sum	15,384	0.6457	1.0000	7692	7692	6154	4614	3077	1538	769

Cluster, population cluster size (N), average of Modified Rogers’ distance per cluster (*mrdMean*), proportion of *mrd* per cluster (*p*), number assigned (*nD*), correction of the assigned number due to the small groups (s50), assigned sample size for 40, 30, 20, 10, and 5% of the population (*s40* to *s05*)

^a^After the correction, due to nD > N, the complementary sample size was reassigned on the other groups proportionally to *mrdMean*

### Diversity analysis

**Fig. 1:**
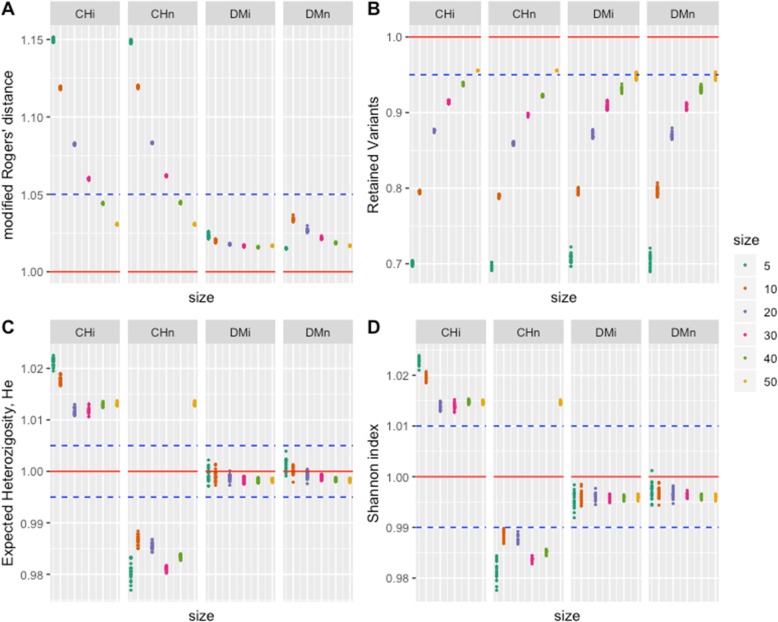
Response of (**a**) Modified Rogers’ genetic distance (*mrd*), **b** number of polymorphic markers (variants, *poly*), **c** Expected Heterozygosity index (genetic diversity index, *he*), and **d** Shannon entropy index (*shan*), for six sample sizes (0, 5, 10, 20, 30, 40 and 50% of the size of the whole collection), two types of samples (independent: i, and nested: n), and two strategies for selecting the best sample (Core Hunter: CH, and D-method: DM). The response is expressed as the (sample value) / (collection value) ratio (red horizontal line). Blue lines represent different sized intervals of the distance from the collection value

**Fig. 2:**
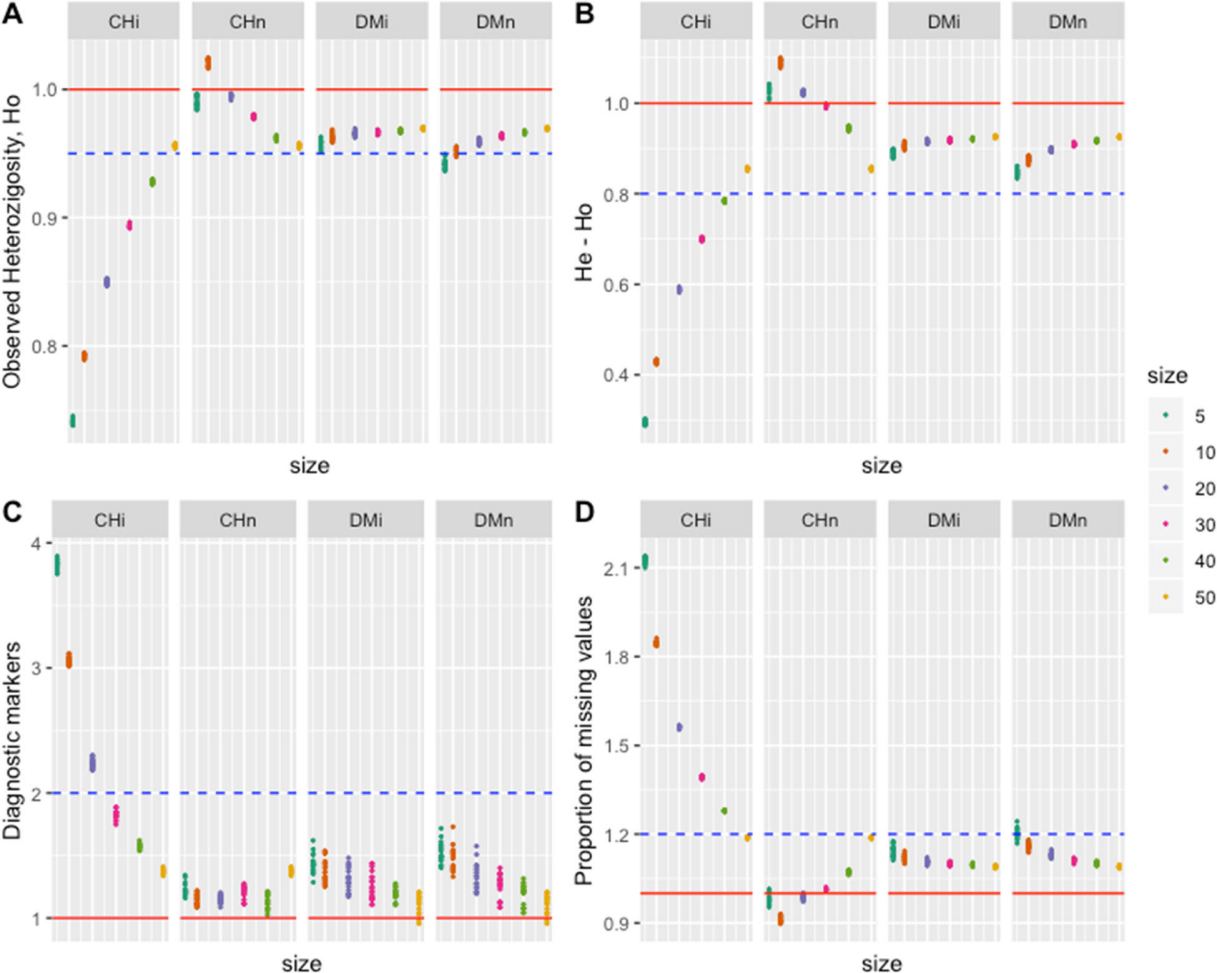
Response of (**a**) Observed Heterozygosity (*ho*), **b** difference of expected minus observed heterozygosity (*he - ho*), **c** number of Diagnostic Markers (markers fixed only for a small number of genotypes in the sample, *ndiag*), and **d**. proportion of missing values in the sample (*pmiss*), for six sample sizes (0, 5, 10, 20, 30, 40 and 50% of the size of the whole collection), two types of samples (independent: i, and nested: n), and two strategies for selecting the best sample (Core Hunter: CH and D-method: DM). The response is expressed as the quotient (sample value) / (collection value) (red horizontal line). Blue lines represent different sized intervals of the distance from the collection value

The eight criteria we used in this study to evaluate the different approaches to panel definition showed different performance with respect to sample size (5 to 50% of the collection size), the type of sampling (independent or nested), and the method for building the core (statistical stratified sampling versus numerical maximization based sampling). Because all the criteria (except for the number of retained variants and diagnostic markers) have a range of possible values between 0 and 1, we also used the ratio of sample value to population value to compare the approaches (red line in Figs. 1 and 2) and evaluate the representativeness of the approaches considering the overall population (Tables 2 and 3).

**Table 2:** Average values for 7 diversity measures characterizing the markers

Method^a^	Sample Size (%)	*pmiss*	*he*	*ho*	*shan*	*mrd*	*Npoly*	*ndiag*
Pop		0.0466	0.0400	0.0655	0.0962	0.1333	161,104	525
CHi	5	0.0989	0.0466	0.0485	0.0984	0.1533	112,843	2004
CHn	5	0.0458	0.0393	0.0649	0.0944	0.1532	112,136	644
DMi	5	0.0532	0.0400	0.0627	0.0958	0.1364	113,974	751
DMn	5	0.0560	0.0401	0.0617	0.0960	0.1353	113,550	797
CHi	10	0.0859	0.0407	0.0519	0.0981	0.1492	128,160	1606
CHn	10	0.0425	0.0395	0.0669	0.0951	0.1493	127,227	600
DMi	10	0.0522	0.0400	0.0630	0.0958	0.1360	128,184	719
DMn	10	0.0540	0.0400	0.0623	0.0959	0.1379	128,252	774
CHi	20	0.0727	0.0405	0.0557	0.0976	0.1443	141,212	1173
CHn	20	0.0459	0.0395	0.0651	0.0950	0.1444	138,439	603
DMi	20	0.0515	0.0400	0.0632	0.0958	0.1357	140,265	687
DMn	20	0.0526	0.0400	0.0628	0.0959	0.1369	140,189	696
CHi	30	0.0649	0.0405	0.0585	0.0976	0.1414	147,391	960
CHn	30	0.0472	0.0393	0.0641	0.0947	0.1416	144,521	634
DMi	30	0.0512	0.0400	0.0633	0.0958	0.1356	146,291	656
DMn	30	0.0518	0.0400	0.0631	0.0959	0.1362	146,273	660
CHi	40	0.0595	0.0406	0.0608	0.0976	0.1392	151,177	825
CHn	40	0.0499	0.0394	0.0630	0.0948	0.1393	148,610	595
DMi	40	0.0511	0.0400	0.0634	0.0958	0.1355	150,016	626
DMn	40	0.0513	0.0400	0.0633	0.0958	0.1358	150,062	623
CHi	50	0.0553	0.0406	0.0626	0.0976	0.1374	153,933	720
CHn	50	0.0553	0.0406	0.0626	0.0976	0.1374	153,933	720
DMi	50	0.0507	0.0400	0.0635	0.0958	0.1356	152,828	587
DMn	50	0.0507	0.0400	0.0635	0.0958	0.1356	152,828	587
SEmin		0.000022	0.000002	0.000011	0.000006	0.000006	11.5	2.3
SEmax		0.000194	0.000017	0.000064	0.000040	0.000039	292.8	12.6

Sampling method (method) and sample size: proportion of missing values (*pmiss*), expected and observed heterozygosity (*he*, *ho*), entropy Shannon index (*shan*), Modified Rogers’ distance (*mrd*), number of retained markers (*npoly*), and number of markers fixed for only a few accessions (*ndiag*). Minimum and maximum values of standard errors of the means (SEmin, SEmax)

^a^*Pop* Population, *CHi* Core Hunter, independent sample, *CHn* Core Hunter, nested sample, *DMi* D-method, independent sample, *DMn* D-method, nested sample, *SE* minimum and maximum standard error of mean

**Table 3:** Sample/population ratio for 7 diversity measures characterizing the markers

Method^a^	Sample size (%)	*pmiss*	*he*	*ho*	*shan*	*mrd*	*npoly*	*ndiag*
CHi	5	2.124	1.021	0.741	1.023	1.150	0.700	3.818
CHn	5	0.984	0.981	0.991	0.981	1.149	0.696	1.227
DMi	5	1.143	0.999	0.957	0.995	1.023	0.708	1.430
DMn	5	1.202	1.001	0.942	0.997	1.015	0.705	1.518
CHi	10	1.846	1.018	0.792	1.020	1.119	0.796	3.060
CHn	10	0.912	0.987	1.021	0.989	1.119	0.790	1.143
DMi	10	1.121	0.999	0.963	0.996	1.020	0.796	1.370
DMn	10	1.159	1.000	0.952	0.997	1.034	0.796	1.475
CHi	20	1.562	1.012	0.850	1.014	1.083	0.877	2.234
CHn	20	0.986	0.985	0.995	0.988	1.083	0.859	1.149
DMi	20	1.106	0.999	0.966	0.996	1.018	0.871	1.309
DMn	20	1.129	0.999	0.959	0.997	1.027	0.870	1.326
CHi	30	1.394	1.012	0.894	1.014	1.060	0.915	1.828
CHn	30	1.013	0.981	0.979	0.984	1.062	0.897	1.209
DMi	30	1.100	0.998	0.966	0.996	1.017	0.908	1.249
DMn	30	1.112	0.999	0.964	0.996	1.022	0.908	1.257
CHi	40	1.279	1.013	0.928	1.015	1.044	0.938	1.571
CHn	40	1.071	0.984	0.962	0.985	1.045	0.922	1.134
DMi	40	1.097	0.998	0.968	0.996	1.016	0.931	1.192
DMn	40	1.101	0.998	0.966	0.996	1.019	0.932	1.187
CHi	50	1.188	1.013	0.956	1.015	1.031	0.956	1.371
CHn	50	1.188	1.013	0.956	1.015	1.031	0.956	1.371
DMi	50	1.090	0.998	0.970	0.996	1.017	0.949	1.119
DMn	50	1.090	0.998	0.970	0.996	1.017	0.949	1.119
SEmin		0.0005	0.00010	0.00020	0.00010	0.00001	0.00010	0.00430
SEmax		0.0042	0.00040	0.00100	0.00040	0.00030	0.00180	0.02410

Sampling method (method) and sample size: proportion of missing values (*pmiss*), expected and observed heterozygosity (*he, ho*), entropy Shannon index (*shan*), Modified Rogers’ distance (*mrd*), number of retained markers (*npol*y), and number of markers fixed for only a few accessions (*ndiag*). Ratio is equal to 1.0 if sample measure is equal to population measure

^a^*CHi* Core Hunter, independent sample, *CHn* Core Hunter, nested sample, *DMi* D-method, independent sample, *DMn* D-method, nested sample, *SE* minimum and maximum standard error of mean

Below we summarize how the sampling processes under study influenced each of the different criteria and by comparing to population level metrics. It should be pointed out that no statistical tests for comparison are presented because the standard errors were too small in comparison with the average values; thus almost all comparison produces very low *p*-values (Tables 2 and 3), even using Generalized Linear Models assuming Beta, Poisson or Binomial distributions for the indices.

#### Modified Rogers’ distance (mrd)

As the basis for the determination of samples across all approaches, *mrd* is a key evaluation metric. The mean *mrd* for all the samples formed is higher than that of the population (Table 3). This is to be expected given that the process of forming samples maximizes *mrd* through the omission of redundant information produced by similar individuals in the overall population. Mean *mrd* values decrease as the sample size increases, producing similar values between the independent and nested samples within the DM and CH sampling approach (Fig. 1a). The sampling approach had strong influence on the mean *mrd*, with CH generating higher values than DM. This is an expected result illustrating CH’s effectiveness in finding a maximum of the objective function (we are looking for samples maximizing the *mrd* average value by reducing redundancies present in population). On the other hand, the DM-method showed values closer to the population values than the CH-method. For CH independent (CHi) and CH nested (CHn) samples, the estimated *mrd* values are close to the population value (within an interval of 5% of the distance from the collection value, blue line) only when the sample size is greater than or equal to s40, while all sample sizes and types of samples are within this interval of distances for the DM-method (Fig. 1a).

#### Number of retained polymorphic markers (poly)

The number of polymorphic markers, an important measure of diversity, performs differently to the other diversity criteria we evaluated. For the largest sample sizes, s50, *poly* reaches approximately 95% of the population value, while for the smaller sample size, such as s5, *poly* is around 70% of that found in the population (Fig. 1b). The relationship between sample size and the retention of polymorphic loci is not linear. At s10 and above, samples retain close to or more than 80% of the loci found in the population, and the increase in retention diminishes as the sample size increases. The patterns of retained polymorphic markers for all four combination of methods (CH and DM) and type of sample (independent or nested) (Chi, CHn, DMi, and DMn are very similar for all sample sizes (Fig. 1b).

#### Expected heterozygosity (he) and the Shannon index (Shan)

The distribution of values for both *he* and *shan* was similar for any one selected approach (Fig. 1c and d). Method CHi reached the highest values of *he* and *shan* for all the sample sizes, while CHn had the lowest values both above and below the population mean. Methods DMi and DMn performed in a similar manner, with values closer to the known population mean. These results are important, as *he* and *shan* are considered very useful measures of genetic diversity; indeed, *he* is often called “genetic diversity” [26, 27]. From the statistical point of view, we could say that the DM-method estimates the population values with more precision than the CH-method, as the former obtains values that are closer to the known population values for both types of samples and for all sample sizes. That is, DM gets a better sampling representation of the population diversity as measured by *he* or *shan*, while the CH method produces samples that overrepresented (CHi) or underrepresent (CHn) the known population values.

#### Observed heterozygosity (ho) and he-ho measures

Observed heterozygosity shows a different pattern than that shown by *he* and *shan*: all but one (s10) of the values of the selected sets are under the population value. CHn, DMi and DMn are closer to the population value for all sample sizes, while CHi values are lower than the above-mentioned values, particularly for sample sizes lower than or equal to s30. The underestimation of *ho* increases when the sample size decreases for all methods except CHn, showing a non-expected performance (Fig. 2a). Again, the DM seems to be always closer (and within the 5% interval) to the observed heterozygosity of the entire collection.

The *he – ho* difference is a measure of inbreeding ($inb=1-\frac{ho}{he}$), resulting in a positive value when there are more homozygous accessions than expected based on the *he* values, that is, more inbreeding than expected, and a negative value when there are less homozygous accessions than expected, that is, less inbreeding than expected. The whole population showed an inbreeding value of 1–0.0655/0.0400 = − 0.64 (Table 2). The *he-ho* measure performs similarly to observed heterozygosity, indicating that there is more variability for *ho* than the *he* variability. Again, *he-ho* is better estimated for CHn, DMi and DMn, and underestimated (suggesting more inbreeding) for CHi, particularly when the sample sizes are less than or equal to s30 (Fig. 2b). In summary, for criteria *ho* and *he-ho*, DMi and DMn are more stable around the values of the entire collection and always within the 5% interval around the population mean than the CHi and CHn sample method.

#### Diagnostic markers (ndiag)

Diagnostic markers are those variants present in only a few accessions in the population or sample, their presence being indicative or diagnostic for those accessions. When studying genetic differences among accessions, a reduction in sample size should produce an increase in this criterion due to the reduction in the total number of genotypes in the sample. Performance for CHn is similar to DMn and DMi, as they show values close to or higher than (less than twice) the population values; however, while CHn does not show a clear change across sample sizes, DMn and DMi decrease when sample size increases (Fig. 2c). In contrast, CHi shows values higher than twice the population value when the sample size is less than s30, indicating strong selection for contrasting allelic germplasm.

#### Proportion of missing values (pmiss)

The proportion of missing values is an important measure, not for diversity per se, but for the quality (completeness) of information represented by a sample. Nearly all samples have higher proportions of missing values than the population (Fig. 2d, see red line). A proportion higher than 1.25 times the value of missing values in the population is obtained for the CHi method when the sample size is less than s30. For the other methods and sample sizes, the proportion of missing values is not greater than 1.25 times the population value. The proportions of missing values in CHn samples increase as sample sizes increase from s10. In contrast, both DM methods show the inverse relationship, that is, the *pmiss* values decrease when sample size increases (Fig. 2d), however the influence of sample size is less marked with values more closely tracking the *pmiss* of the entire collection.

#### How the samples are selected: multidimensional scaling 2D graphical representation for the entire collection

**Fig. 3:**
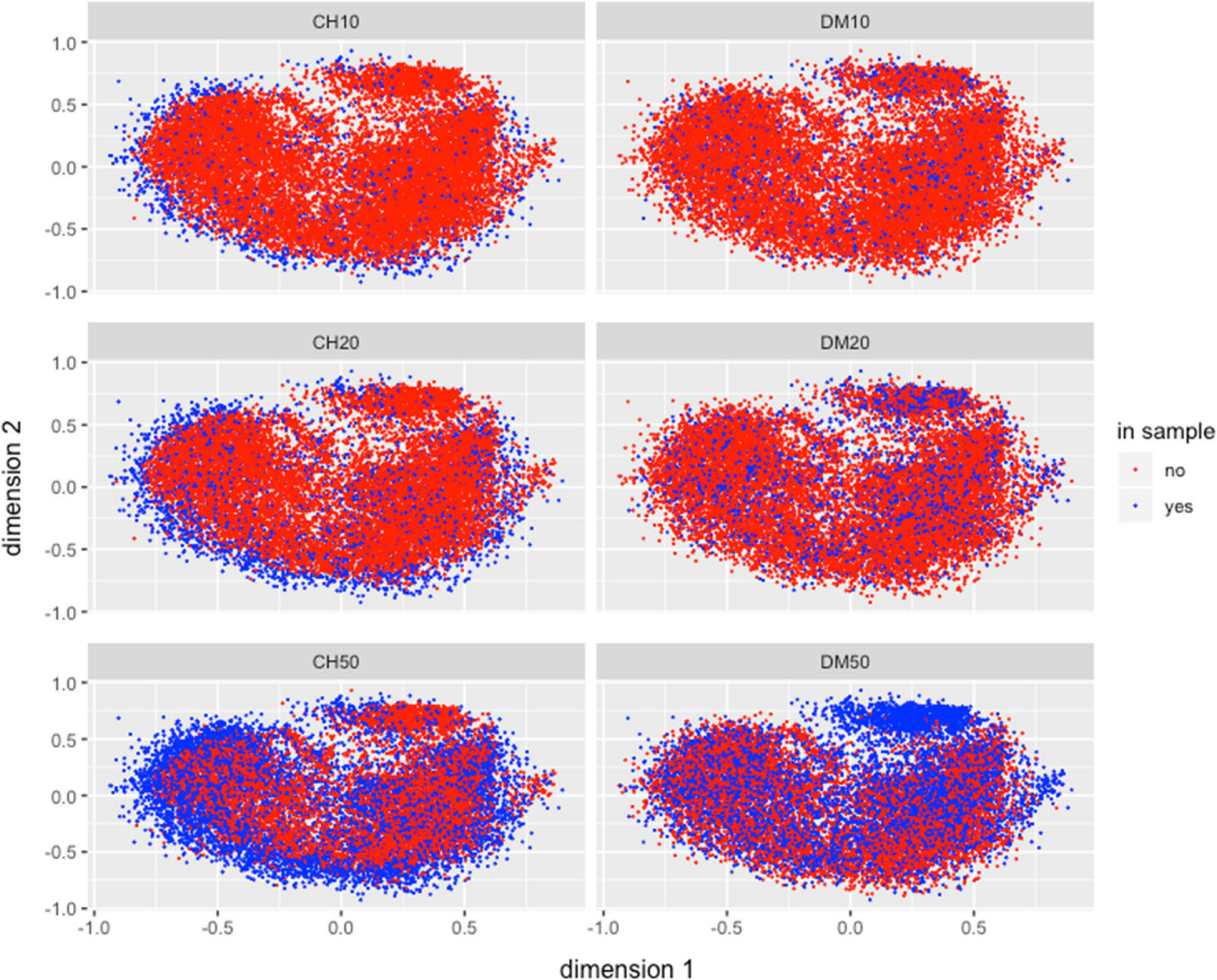
Multidimensional Scaling graphical representation in two dimensions of the Modified Rogers’ genetic distance (*mrd*) between pairs of genotypes in the best independent samples of sizes 10, 20, and 50% of the entire collection. The red points correspond to the whole collection, the blue points denote genotypes selected for the two strategies Core Hunter = CH and D-method = DM

The observed differences between the CH and DM methods with respect to diversity measures and sampling representativeness, could be better understood by observing the accessions being selected by the CH and DM methods for different sample sizes in the multidimensional scaling representation of the mrd in two dimensions. Figure 3 shows the best independent s10, s20 and s50 samples selected from the collection by both methods; it illustrates that the s10 sample from CH captured more the diversity from the borders of the entire collection, while the sample from DM captured genotypes distributed across the population (blue dots). The same behavior of the sample is observed for the other sample sizes, s20 and s50. Method CH maximizes diversity by sampling the extreme accessions of the entire collection, whereas DM method uniformly samples all parts of the entire collection. Similar results are found for s20 and s50 (Fig. 3) where samples from the DM method gave a more uniform representation of the distribution of accession in the entire collection of 15,384 maize accessions.

**Fig. 4:**
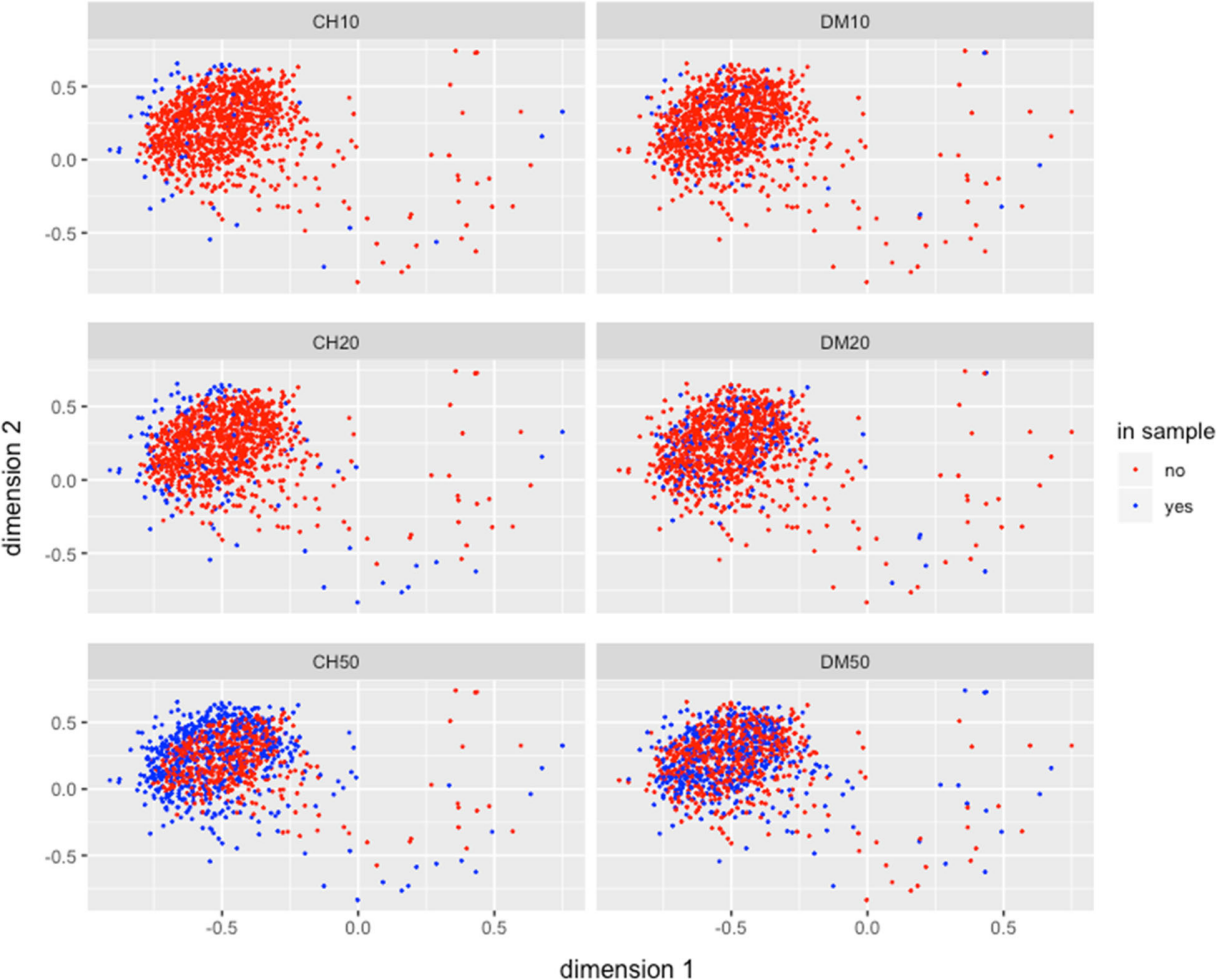
Multidimensional Scaling graphical representation in two dimensions of the Modified Rogers’ genetic distance (*mrd*) between pairs of genotypes in the best independent sample sizes of 10, 20, and 50% of the Conico maize race collection. The red points correspond to the whole collection, the blue points denote genotypes selected for the two strategies Core Hunter = CH and D-method = DM

The two dimensional representation of the multidimensional scaling of the mrd for the accessions selected for independent samples s10, s20, s50 by CH and DM for the maize race Conico is shown in Fig. 4. Similar to results already described for the case considering the entire collection (Fig. 3), the DM method gave a much more complete representation of the total variability existing in the Conico maize race than the CH method that concentrates the sampling at the extreme of the distribution of Conico accessions for the three independent samples sizes (s10, s20 and s50)**.**

**Fig. 5:**
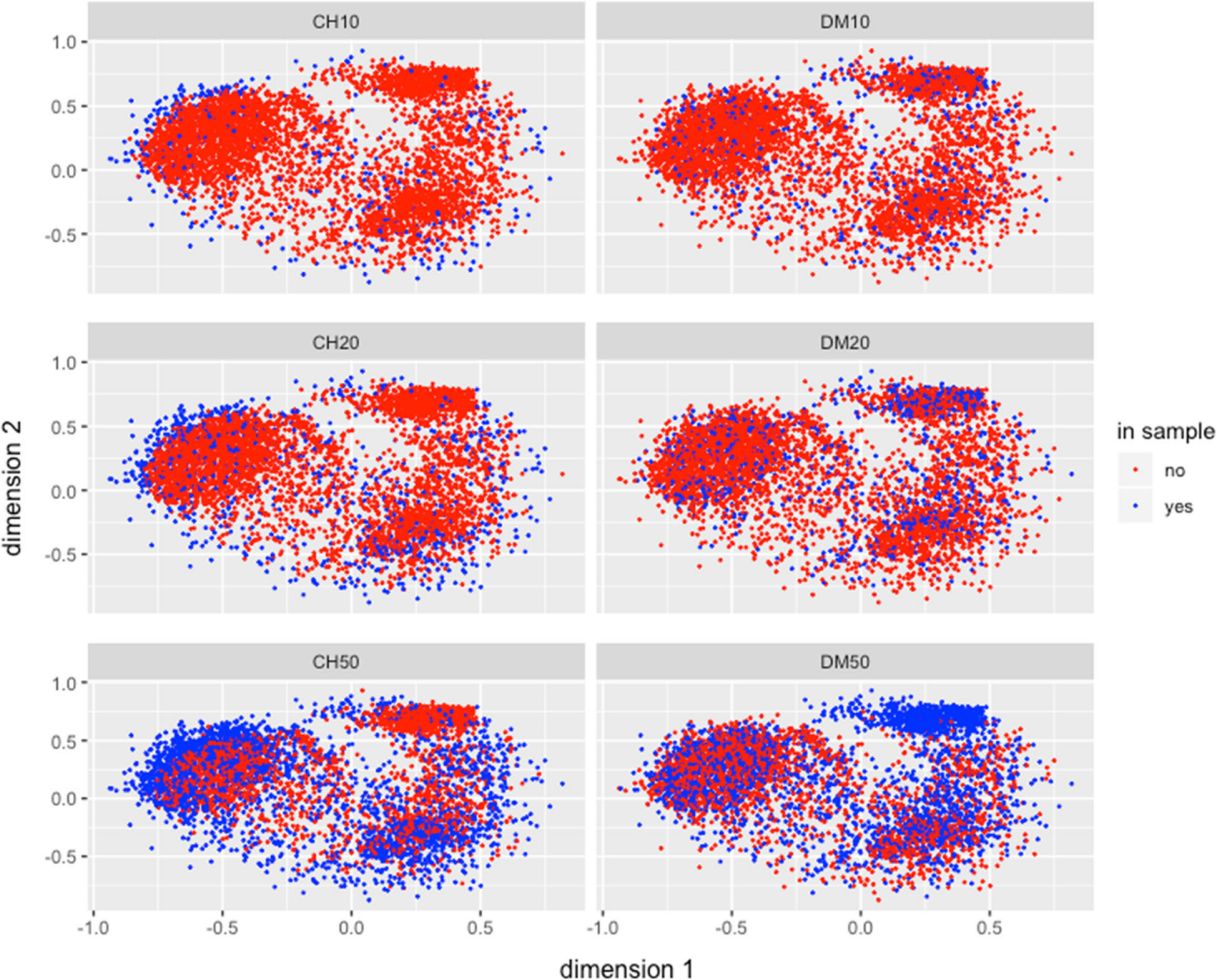
Multidimensional Scaling graphical representation in two dimensions of the Modified Rogers’ genetic distance (*mrd*) between pairs of genotypes in the best independent samples sized 10, 20, and 50% of the accession collected in the Adaptation Area HIGHLANDS of the collection. The red points correspond to the whole collection, the blue points denote genotypes selected for the two strategies Core Hunter = CH and D-method = DM

Furthermore, when examining the two dimensional representations of the multidimensional scaling of the mrd for accessions of maize selected in the highland adaptation zone (Fig. 5), using independent samples of sizes S1, S20 and S50, we observed again that samples from DM methods are more representative than those maize accessions selected by the CH methods that concentrate the selection of samples more at the borders of the distribution. In summary, for the entire maize collection and for samples based o race and adaptation, the Conico maize and highland maize samples respectiveyl, CH basically selects at the extreme borders of diversity distributions, whereas the DM selects accessions across the whole spectrum of diversity.

#### Stability: variability among repetitions of the same process

Table 4 shows changes in the variability among repetitions (20 repetitions) within each method, type of sample and sample size; to be clear we present the ratio of standard deviations: stdev_sample / stdev_s50. The most important result is that low sample sizes imply more variability among repetitions, that is, the probability of obtaining a “bad” (or a “very good”) sample increases inversely to the sample size. A second observation is that CHi generates more similar repetitions than other methods and types of samples (independent or nested) for *pmiss*, *shan*, *polymorphic markers* and *specific markers*, while DMi generates more similar repetitions for *he*, *ho*, and *mrd* criteria; a third observation is that the most unstable criteria, that is, the criteria showing more different repetitions, are *poly* and n*diag*.

**Table 4:** Variability among 20 repetitions

Method	size	*pmiss*	*he*	*ho*	*shan*	*mrd*	*variants*	*ndiag*
CHi	50^a^	1.097	0.122	0.624	0.267	0.499	51.185	10.058
CHi	40	0.9	1.1	1.1	1.2	1.1	5.0	1.1
CHi	30	1.4	2.0	1.3	2.2	1.3	5.7	1.9
CHi	20	1.6	2.1	1.7	2.4	1.2	3.3	1.8
CHi	10	2.9	2.3	1.7	2.3	1.8	3.5	1.5
CHi	5	4.3	2.6	2.0	2.6	2.8	5.6	2.2
CHn	40	1.8	1.2	1.4	1.2	1.0	3.6	3.0
CHn	30	1.5	1.2	1.2	1.4	0.9	4.1	2.6
CHn	20	2.7	2.4	1.4	2.5	0.8	4.1	1.5
CHn	10	4.2	3.3	2.9	3.4	1.6	5.3	2.1
CHn	5	7.0	6.3	4.6	6.8	2.2	9.8	2.8
method	size	*pmiss*	*he*	*ho*	*shan*	*mrd*	*variants*	*ndiag*
DMi	50^a^	1.512	0.099	0.504	0.327	0.363	559.521	39.740
DMi	40	1.1	1.3	1.1	1.1	1.0	0.8	0.6
DMi	30	1.5	2.0	1.6	1.7	1.4	1.2	1.4
DMi	20	2.1	2.5	2.1	2.2	1.4	1.1	1.2
DMi	10	3.1	4.4	3.5	3.5	3.0	0.9	1.2
DMi	5	5.7	5.2	4.1	4.7	4.8	1.8	1.0
DMn	40	1.4	1.0	1.2	0.9	1.1	0.9	1.1
DMn	30	1.7	1.5	1.4	1.3	2.0	1.0	1.3
DMn	20	2.5	2.7	2.3	2.4	3.5	1.1	1.4
DMn	10	3.2	3.3	3.2	3.3	3.9	1.4	1.3
DMn	5	5.3	4.6	4.5	4.6	0.8	2.3	1.1

Standard deviation for samples (s5, s10, …, s40) expressed as the ratio in respect to standard deviation from independent samples size 50%

^a^In independent samples, size = 50, values are the standard deviations multiplied by 10^4^, and other values are sSample/50Sample ratios

#### Recovery of external information (races and adaptation areas)

Tables 5 and 6 show the classification of accessions in the collection for 23 races and for the 8 adaptation areas where the accessions were collected. A good sample from that population should select, for each race and area, proportions similar to the proportion in the collection. The last two columns in Tables 5 and 6 show the discrepancies in the proportion of each race and area in the collection vs. the samples obtained by the CH and DM methods. For race recovery, the maximum values of discrepancy were 2.63 and 1.76% for the CH and DM methods, respectively, while for the adaptation areas, the maximum values were 4.62 and 3.70%, respectively. The DM method showed a slight advantage, but overall both approaches selected appropriate proportions of both external (to the analysis) variables.

**Table 5:** Recovery of races from the collection

Race	nobs	Proportion			abs (diff)	
		Collection	CH	DM	CH	DM
AVMORO	277	0.0180	0.0052	0.0208	0.0128	0.0028
BOLITA	253	0.0164	0.0033	0.0182	0.0132	0.0018
CELAYA	290	0.0189	0.0202	0.0189	0.0013	0.0000
CHALQU	364	0.0237	0.0358	0.0241	0.0121	0.0004
COMITE	101	0.0066	0.0104	0.0033	0.0038	0.0033
CONICO	1135	0.0738	0.0475	0.0592	0.0263	0.0146
CONNOR	488	0.0317	0.0455	0.0234	0.0138	0.0083
CRISCO	210	0.0137	0.0091	0.0312	0.0045	0.0176
DENBLA	132	0.0086	0.0046	0.0137	0.0040	0.0051
DENTAD	293	0.0190	0.0039	0.0124	0.0151	0.0067
DTRGRU	222	0.0144	0.0026	0.0117	0.0118	0.0027
ELOTCO	137	0.0089	0.0111	0.0104	0.0021	0.0015
NALTEL	218	0.0142	0.0169	0.0046	0.0027	0.0096
OLOTIL	149	0.0097	0.0065	0.0072	0.0032	0.0025
OLOTON	252	0.0164	0.0215	0.0085	0.0051	0.0079
PADENT	433	0.0281	0.0137	0.0293	0.0145	0.0011
PEPITI	125	0.0081	0.0111	0.0078	0.0029	0.0003
RGDENT	156	0.0101	0.0104	0.0104	0.0003	0.0003
SALVAD	223	0.0145	0.0026	0.0065	0.0119	0.0080
SAPEPY	104	0.0068	0.0020	0.0052	0.0048	0.0016
TABLON	224	0.0146	0.0286	0.0104	0.0140	0.0042
TEPECI	175	0.0114	0.0156	0.0059	0.0042	0.0055
TUXPEN	795	0.0517	0.0553	0.0377	0.0036	0.0140
Sum or Mean	15,384	1.0000	1.0000	1.0000	0.0082	0.0052
NOID	8628	0.5608	0.6170	0.6196	0.0562	0.0588
sum	15,384	1.0000	1.0000	1.0000	0.2445	0.1785

Number of accessions belonging to the main identified races (nobs); proportion of accessions belonging to each race in the whole collection and recovered for the sampling methods (CH, DM); absolute value of the discrepancy between the sample recovered proportion and the collection proportion (abs (diff)). Including and not including non-identified (NOID) races

**Table 6:** Recovery of adaptation areas from the collection

Adapt	nobs	Proportion			Abs (diff)	
		Collection	CH	DM	CH	DM
DRL	1240	0.0806	0.0852	0.0611	0.0046	0.0195
DRM	2289	0.1488	0.1333	0.1437	0.0155	0.0051
HIG	4922	0.3199	0.2737	0.3563	0.0462	0.0364
LOW	167	0.0109	0.0156	0.0143	0.0047	0.0034
MID	322	0.0209	0.0228	0.0286	0.0018	0.0077
WLM	1179	0.0766	0.0735	0.0546	0.0032	0.0220
WLO	2440	0.1586	0.2042	0.1216	0.0456	0.0370
WUM	2053	0.1335	0.1502	0.1203	0.0173	0.0180
Sum or mean	14,612	0.9498	0.9584	0.9005	0.1383	0.1443
NOI	772	0.0502	0.0416	0.0995	0.0086	0.0493
Sum	15,384	1.0000	1.0000	1.0000	0.1469	0.1936

Number of accessions belonging to the main identified adaptation areas (nobs); proportion of accessions belonging to each adaptation area in the whole collection and recovered for the sampling methods (CH, DM); absolute value of the discrepancy between the sample recovered proportion and the collection proportion (abs (diff)). Including and not including the non-identified (NOI) areas, dry lowland (DRL), dry mid-altitude (DRM), highland (HIG), lowland (LOW), mid-altitude (MID), wet lower mid-altitude (WLM), wet lownand (WLO), wet upper mid-altitude (WUM)

Finally, Table 7 shows the most used genetic diversity measures and the Wright (1951) statistics for race and adaptation based samples. The table also shows the collection (population) values and their estimation by CH and DM methods (best s20 samples). Sample estimated values are similar to the collection values; this is an interesting point particularly for the F_ST_ statistic measuring the proportion of expected heterozygosity explained by the differences among groups (AMOVA Fst): the differences among adaptation areas (2.67% for population, 2.89% for the DM sample and 1.92% for the CH sample) and races (7.89, 8.48 and 8.38% for population, DM sample, and CH sample, respectively). In general, DM is closer to the collection (population) values than CH. However, both strategies gave good estimates of the differences among races and areas, as compared with the collection studied.

**Table 7:** Diversity measures in the collection and in the best independent samples s20 (20% of collection)

	Adaptation			Races		
	Collection	DM	CH	Collection	DM	CH
H_O_	0.0655	0.0632	0.0555	0.0655	0.0632	0.0555
H_T_	0.0400	0.0400	0.0405	0.0400	0.0400	0.0405
H_S_	0.0390	0.0389	0.0397	0.0369	0.0366	0.0371
H_B_	0.0011	0.0012	0.0008	0.0032	0.0034	0.0034
F_ST_	0.0267	0.0289	0.0192	0.0789	0.0848	0.0838
F_IT_	−0.6356	−0.5801	−0.3700	−0.6356	−0.5801	−0.3700
F_IS_	−0.6805	−0.6271	−0.3967	−0.7756	− 0.7266	− 0.4953

DM-method and CH-method; groups of adaptation and race; mean of observed heterozygosity (Ho); mean of expected heterozygosity (H_T_), average of within group (H_S_) and between groups (H_B_) expected heterozygosity and H_B_/H_T_ ratio (AMOVA-F_ST_). Wright statistics F_IT_ (proportional deviation of observed from expected heterozygosity in the whole collection), F_IS_ (proportional deviation of observed from expected heterozygosity within groups of adaptation or race)

## Discussion

### Sample size effects

The strongest effect of sample size was observed for the CHi method on heterozygosity (*he* and *he-ho* indices), number of diagnostic markers (*ndiag*) and proportion of missing values in the sample (*pmiss*); for those criteria, the differences were greater when the sample size decreased. For the other methods, the effect of sample size reduction did not have the same strong effect. Samples of sizes greater than s20 retained more than 85% of the polymorphic markers for all methods and types of samples. In all cases, a reduction in sample size was associated with an increase in the standard deviation among repetitions, that is, the processes were more unstable.

### Type of sample effects

Independent and nested samples showed similar performance with respect to all the criteria for the DM-method. In contrast, there are differences between nested and independent samples obtained by the CH-method, particularly for expected and observed heterozygosity, Shannon index, number of diagnostic markers and proportion of missing values in the sample. In these cases, nested samples performed better than independent samples and were more stable for the different criteria. This finding is of value as the use of nested samples, avoiding the selection of very different accessions for different sample sizes, is of benefit to collection managers as efforts can be focused on maintaining sufficient seed/clones of a defined sub-set of the collection for more frequent distribution to clients.

### Strategies for selecting samples (CH, DM methods)

Results in this paper show that for all the criteria (except the number of retained polymorphic markers, but including the *mrd* genetic distance used as the objective function), for all sample sizes, and for both types of samples (independent or nested), the statistical DM method gives a better approximation to the known population values (that is, the sample/population ratio is closer to one) than the CH method. This result was expected, as the main strength of the statistical stratified random sampling strategy consists of giving to individuals from the same stratum (group or cluster based on *mrd* distance) the same probability to be selected into the sample, selecting any of them at each step of the sampling process, and assigning to each group a sample size proportional to its diversity. When used to build nested samples, the CH method produces similar results as the DM, but shows different results for the most important criteria, genetic diversity (*he*) and Shannon index (*shan*), both of which are underestimated.

Differences between the CH and DM approaches to obtain samples were observed in the Multidimensional Scaling 2D-representation of the collection, and the best selected (by CH and DM) independent s10, s20 and s50 samples were compared. While these representations illustrate advantages of the DM-method over the CH-method in terms of representativeness appeared during the selection process, they also shows a possible weakness of the DM-method: when a group (cluster obtained in the first stage for DM) shows high diversity, the method selects a large number of genotypes for the sample; if the group size is not big enough, the method could select all or almost all the group genotypes (see the upper-right cloud of blue dots in Fig. 3, DM50, and compare it to the assigned number of genotypes selected from groups 4 and 6 by the DM-method in Table 1**)**. In summary, both strategies (CH, DM) could be used simultaneously to obtain the advantages and avoid the weakness of each.

## Conclusions

The representativeness and genetic diversity found by this study in a large number of maize accessions from the CIMMYT germplasm bank show a stronger effect on sample size with the CH method than with the DM method. Sample sizes greater than 20% of the total size of the populations retained more than 85% of the polymorphic markers with both the CH and the DM methods. Independent and nested samples showed similar performance with respect to all the criteria for the DM method, but there were differences between nested and independent samples obtained by the CH method.

In general, for most of the criteria, the statistical DM method achieved better approximations to the known population values than the CH method. The plot of the first two multidimensional scaling dimensions of the collection and the best (out of 20 repetitions) sample selected by CH and DM for independent samples of sizes from 10 to 50% clearly shows the biases in the core sample selected by the CH method, compared with the more complete, less biased and more uniform core sample selected under the statistical DM. In terms of comparing both sampling methods for recovering the information on races and their areas of adaptation, the results favored the DM method over the CH method for better recovering the information existing in the entire collection.

## Methods

### Genotype germplasm bank accessions

We worked with data from an initial genotyped collection of 22,903 germplasm bank accessions from CIMMYT’s germplasm bank, material available for distribution under the Standard Material Transfer Agreement (SMTA) of the International Treaty on Plant Genetic Resources for Food and Agriculture (http://www.fao.org/3/a-bc083e.pdf). These landraces were genotyped with DArTseq™ technology. The genotyping was conducted on composite samples (30 individuals) represented in each accession DNA sample. A total of 616,967 biallelic single nucleotide polymorphism (SNP) markers was identified. The frequency of SNP alleles within each sample was determined from the number of sequence counts for each allele. The resulting data were filtered for the presence of missing values (allowing only a maximum of 20%) and marker coverage (greater than 2.0) to develop a final dataset of 161,104 SNP markers. The germplasm was filtered to a final set of 15,384 maize landraces, the availability of geographic data from collection site origins being used as a selection criteria. Table 8 describes racial and adaptation composition of the panel of 15,384 landraces, Table 9 shows the characteristics of the markers finally used on the 15,384 maize accessions. All data used in the final analysis along with associated identifiers, descriptions of accessions used and marker filtering parameters are available via The CIMMYT Seeds of Discovery repository of the CIMMYT Research Data and Software Repository Network (https://data.cimmyt.org/dataverse/seedsofdiscoverydvn) under a study entitled “SNP Allele Frequencies and Descriptive Data of 15,384 CIMMYT Germplasm Bank Maize Landrace Accessions”, http://hdl.handle.net/11529/10548315. This data is available under the license and terms of use described in http://hdl.handle.net/11529/10548315 in alignment with germplasm availability under the SMTA.

**Table 8:** Structure of dataset

Races										Adaptation				
Race	Nobs	collection	CH	DM	Race	nobs	collection	CH	DM		Nobs	collection	CH	DM
AVMORO	277	0.0180	0.0052	0.0208	NALTEL	218	0.0142	0.0169	0.0046	DRL	1240	0.0806	0.0852	0.0828
BOLITA	253	0.0164	0.0033	0.0182	OLOTIL	149	0.0097	0.0065	0.0072	DRM	2289	0.1488	0.1333	0.1494
CELAYA	290	0.0189	0.0202	0.0189	OLOTON	252	0.0164	0.0215	0.0085	HIG	4922	0.3199	0.2737	0.3159
CHALQU	364	0.0237	0.0358	0.0241	PADENT	433	0.0281	0.0137	0.0293	LOW	167	0.0109	0.0156	0.0105
COMITE	101	0.0066	0.0104	0.0033	PEPITI	125	0.0081	0.0111	0.0078	MID	322	0.0209	0.0228	0.0201
CONICO	1135	0.0738	0.0475	0.0592	RGDENT	156	0.0101	0.0104	0.0104	NOI	772	0.0502	0.0416	0.0447
CONNOR	488	0.0317	0.0455	0.0234	SALVAD	223	0.0145	0.0026	0.0065	WLM	1179	0.0766	0.0735	0.0791
CRISCO	210	0.0137	0.0091	0.0312	SAPEPY	104	0.0068	0.0020	0.0052	WLO	2440	0.1586	0.2042	0.1627
DENBLA	132	0.0086	0.0046	0.0137	TABLON	224	0.0146	0.0286	0.0104	WUM	2053	0.1335	0.1502	0.1349
DENTAD	293	0.0190	0.0039	0.0124	TEPECI	175	0.0114	0.0156	0.0059					
DTRGRU	222	0.0144	0.0026	0.0117	TUXPEN	795	0.0517	0.0553	0.0377					
ELOTCO	137	0.0089	0.0111	0.0104	NOI	8628	0.5608	0.6170	0.6196					
sum						15,384	1.0000	1.0000	1.0000		15,384	1.0000	1.0000	1.0000

Races: number (nobs) of accessions from 23 main identified plus one subset of 10 other minor and non-identified (NOI)

Adaptation: number of accessions from nine areas where accessions were collected plus non-identified areas (NOI), dry lowland (DRL), dry mid-altitude (DRM), highland (HIG), lowland (LOW), mid-altitude (MID), wet lower mid-altitude (WLM), wet lownand (WLO), wet upper mid-altitude (WUM). Proportion in the whole collection and in independent samples sized 20% of the collection. Core Hunter (CH) and DM methods

**Table 9:** Data description^a^ of the whole collection (population)

	*pmiss*	*nefgen*	*pest*	*nhom*	*ho*	*he*	ae	*shan*
Minimum.	0.0000	12,308	0.0000	701	0.0000	0.0000	1.0000	0.0000
1st Quantile	0.0018	14,183	0.9970	13,285	0.0007	0.0002	1.0000	0.0013
Median	0.0190	15,092	0.9997	14,674	0.0031	0.0005	1.0010	0.0035
Mean	0.0466	14,668	0.9492	13,698	0.0655	0.0400	1.0630	0.0962
3rd Quantile	0.0781	15,357	0.9999	15,234	0.0188	0.0050	1.0050	0.0251
Maximum	0.1999	15,384	1.0000	15,383	0.9544	0.5000	2.0000	1.0000

^a^Proportion of missing values (*pmiss)*, number of accessions showing information (*nefgen)*, allele frequency estimation (*pest*), number of homozygous accessions (*nhom*), observed heterozygosity (*ho*), expected heterozygosity (*he)*, number of effective alleles (*ae*), Shannon entropy index (*shan*)

### Sampling methods

#### Three stage stratified random sampling: the D-method (DM)

Briefly the D-method [13, 16, 17] begins by classifying the accessions into groups (clusters) based on the Modified Rogers’ genetic distance (*mrd*) [28] using the “minimum variance within groups” clustering method, as proposed by [29]. The appropriate number of groups is defined graphically, using the between and within sum of squares and their related “pseudo F” statistic. The number of accessions to be selected from each cluster is then defined proportionally to the mean *mrd* of each cluster. After defining the number of accessions to sample from each cluster, a thousand independent stratified random samples are obtained and the mean *mrd* values for each are calculated; the sample showing the maximum mean *mrd* value overall is selected as the optimal germplasm panel.

#### Core hunter 3: the CH-method

As described in [25] “Core Hunter is a multi-purpose core subset selection tool that uses local search algorithms to generate subsets relying on one or more metrics, including several distance metrics and allele richness.” It is implemented in the R [30] package Core Hunter (http://www.Core Hunter.org, reviewed October 2018) and allows the user to define and use different options. In this work we applied the CH-method to an *mrd* distance matrix and the default options. As described in [25] “Core Hunter 3 constructs core collections with high diversity (high entry-to-nearest-entry distance; E-NE) and which maximally represent the individual accessions from the entire collection.”

### Sampling process

The nested and the independent sample represents two methods to provide users of the germplasm bank sample of accessions. Independent denotes that every time a new sample is taken is independent from the previous ones; nested denotes when a big sample of the collection is taken and then subsamples from the big original sample are taken for full filing user’s demands for that accession. Twenty repetitions of nested and independent samples sized 50, 40, 30, 20, 10, and 5% from the entire collection (s50 = 7692, s40 = 6154, s30 = 4165, s20 = 3077, s10 = 1538, and s5 = 769 accessions, respectively) were selected using the two previously mentioned sampling methods.

### Diversity measures

Genetic diversity is usually studied from two points of view: allelic genetic diversity, the point of view of geneticists and taxonomists, and between individuals’ genetic diversity, the point of view of breeders [20]. We used allele frequencies to produce six diversity indices: expected and observed heterozygosity and their difference (inbreeding coefficient), Shannon entropy index, number of polymorphic alleles or markers, and diagnostic markers (markers being specific only for a few accessions in the collection). From the “breeder perspective,” we used the *mrd* genetic distance between pairs of individuals, and, finally, the proportion of missing values in the sample as a measure of information recovery. The following diversity criteria were used.

- ***Expected Heterozygosity*** [26], or gene diversity [27], *he*, is the most used index. It is defined as: $0\le {he}_i=1-\sum \limits_{j=1}^2{\hat{p}}_{ij}^2\le 0.5$, for an *i*^*th*^ diploid marker (locus), and $he=\frac{1}{L}\sum \limits_{i=1}^L{he}_i$, the average over all loci, for the population. The index summarizes genetic variation and reaches a maximum value of 0.5 for diploid loci when both allelic frequencies are equal to 0.5, maximum locus diversity.
- ***Observed heterozygosity***, *ho*_*i*_, is the proportion of heterozygotes at locus *i*, and is averaged for population characterization, *ho*. It is affected by inbreeding and other evolutionary processes and then, when compared against *he*, produces the inbreeding coefficient *f* for a locus: *f*_*i*_ = 1 − *ho*_*i*_/*he*_*i*_, and their average value for a population. The *f* coefficient is the maximum likelihood estimator of inbreeding under Hardy-Weinberg equilibrium [27]. We used the *he – ho* difference as a measure of inbreeding: negative values imply high inbreeding, positive values low inbreeding and zero no-inbreeding.
- ***Shannon diversity index*** for the *i*^*th*^ locus: $0\le {sh}_i=-\sum \limits_{j=1}^2{\hat{p}}_{ij}\bullet {\mathit{\log}}_2\left({\hat{p}}_{ij}\right)\le 1$, and its average value for a population. We used the logarithm base 2, because when the allele frequencies are equal to 0.5 the index value is 1.0, maximum of diversity.

#### Modified Rogers’ distance - between individuals genetic distance

Based on its good mathematical and genetic properties [28], we selected the *mrd* between two individuals *x, y*, measured by a set of L SNP markers:

$$ 0\le {mrd}_{xy}=\frac{1}{\sqrt{2L}}\sqrt{\sum \limits_{i=1}^L\sum \limits_{j=1}^2{\left({\hat{p}}_{ijx}-{\hat{p}}_{ijy}\right)}^2}\le 1 $$

Data processing was performed using scripts specifically written for the free software R [30]. A High-Performance Computer containing four nodes, each one formed by 94 Cores and 512 Gb of RAM memory, was used.

#### Number of retained polymorphic markers

The reduction in the number of genotypes generated by the sampling processes could reduce the polymorphism of some markers due to the selection in the sample of genotypes showing the same genetic structure; the opposite result is not possible because a marker that is monomorphic in the collection will continue being monomorphic in any extracted sample. Since the number of polymorphic markers is a measure of a collection’s diversity, we considered its reduction in the samples as a measure of the effect of sampling on measured diversity.

#### Diagnostic markers

When an allele is fixed for only a few genotypes in the collection, we define that allele (or marker) as a diagnostic one, because it identifies and differentiates a few sets of genotypes from the rest of the collection. We observed the performance of those alleles (markers) across different sample sizes and methods.

#### Proportion of missing values

The proportion of missing values in a collection or sample is not a measure of its genetic diversity, but it is a measure of the quality of any statistical set of data: processes that produce low proportions of missing data are better processes.

### Representativeness

From the point of view of statistics, the most important objective when sampling a population is the “sample representativeness”: a good sample should represent the population in terms of the values of the measured traits and the frequency distribution of individuals in the population. Those principles, when applied to genetic diversity, where the measured “traits” are a sample of loci from the genetic structure, imply that a good sample must be a subset of individuals representing most of the genetic structure of the population, that is, the measured and the non-measured loci in the population. One way we can measure the representativeness of a genetic sample is to compare a set of criteria associated with diversity, between the population and the samples, being better the sample that gave rise to values nearer to the known values in the population.

### Stability or repeatability of process

Another important characteristic of a sampling method is its stability or repeatability. In this paper we repeated 20 times each “strategy – type of sample – sample size” combination to measure the repeatability of the sampling processes. The standard deviations between repetitions were calculated for each criterion to obtain a measure of repeatability; when the standard deviation is lower, the process is considered more repeatable and stable.

### Recovery of external information (races and adaptation areas)

Germplasm bank genotypes are characterized for different external (non-genetic) variables. In this case, we found two such variables: genotype race and climatic adaptation area where the genotype was collected. When the sampling process is applied, we expect the external variables to maintain the proportion of genotypes belonging to each external group as in the collection. The proportion of genotypes per variable (race or area) were calculated from the whole collection and from the best s20 independent samples for both methods (CH, DM). They were then compared and the absolute value of discrepancies (population – sample) was used as a measure for the capacity to recover external characteristics. Finally, we conducted an analysis of molecular variance (AMOVA) and calculated Wright [31] statistics to compare the relative performance of each approach in the development of samples with close representation of the overall population.

### Data processing

We used scripts specifically written for the free software R [30], Figures were done using the package ggplot2 [32]. Processes were run in as a high-performance computer containing five nodes, each one formed by 94 cores and 512 Gb of RAM memory.

## Data Availability

The datasets supporting the conclusions of this article along with associated identifiers are available via The CIMMYT Seeds of Discovery repository of the CIMMYT Research Data and Software Repository Network (https://data.cimmyt.org/dataverse/seedsofdiscoverydvn) under a study entitled “SNP Allele Frequencies and Descriptive Data of 15,384 CIMMYT Germplasm Bank Maize Landrace Accessions”, http://hdl.handle.net/11529/10548315. This data is available under the license and terms of use described in http://hdl.handle.net/11529/10548315 in alignment with germplasm availability under the SMTA of the International Treaty on Plant Genetic Resources for Food and Agriculture.

## Abbreviations

CH: Core Hunter strategy
CHi, CHn: independent or nested sample from CH method
DM: Stratified random sampling with size assignation proportional to diversity strategy
DMi, DMn: independent or nested sample from DM method
*he, ho*: expected and observed heterozygosity
*mrd*: Modified Roger Genetic distance
*ndiag*: Number of “diagnostic markers”
*pmiss*: Proportion of missing values
*poly*: Number of polymorphic markers
*shan*: Shannon entropy index
SNP: Single Nucleotide Polymorphism
